# Seasonal variation in fertility and milk production in dairy cattle under Mediterranean climate

**DOI:** 10.3389/fvets.2026.1823940

**Published:** 2026-05-28

**Authors:** Julien S. K. D'hallewin, Babak Darabighane, Adele Frau, Roberta Cresci, Chiara Cosseddu, Sebastiano Sale, Daniela Bebbere, Alberto S. Atzori, Francesca Mossa

**Affiliations:** 1 Department of Veterinary Medicine, University of Sassari, Sassari, Italy; 2 Department of Agriculture, University of Sassari, Sassari, Italy; 3 Independent Practitioner, Dorgali, Italy

**Keywords:** conception rate, dairy cattle, fertility, heat stress, milk composition, milk production, services per conception

## Abstract

Heat stress affects fertility and productivity in dairy cattle, yet field studies from the Mediterranean area remain limited. This retrospective, observational cohort study aimed to investigate seasonal variation in: (1) conception and pregnancy rates (at 30 and 60d post-AI, respectively), number of services per conception, submission rate, calving to conception interval, and gestation length; (2) milk production and composition in Holstein Friesian cattle in two Italian farms during three consecutive years. Reproductive data, milk production, and composition were analyzed both monthly and based on three categories: Minimum risk temperature-humidity index (THI) < 56, Low risk (56 < THI < 68), and High risk (THI> 68). High, Low, and Minimum risk conditions occurred for approximately 4 months/year. In heifers that were not exposed to cooling system, the monthly submission rate peaked in May (17.9%) and was the lowest in September (9.4% *P* < 0.001); also, a greater submission rate was recorded under Minimum and Low risk compared to High risk (*P* < 0.001). In lactating cows which were exposed to cooling systems when THI exceeded 68, conception rate at 30d post-AI, pregnancy rate at 60d post-AI, number of services per conception, monthly submission rate were influenced by month (*P* < 0.001). Under High risk conception rate at 30d post-AI (High risk = 37.2%; Minimum risk = 55.2%; *P* < 0.05), pregnancy rate at 60d post-AI (High risk = 29.4%, Minimum risk = 45.8%; *P* < 0.05) and submission rate (High risk = 13.1%, Minimum risk = 17.9%; *P* < 0.001) were reduced compared with Minimum risk. Milk production peaked in spring and decreased in summer and was lower under High (10.90 ton/day) than Minimum risk (11.91 ton/day; *P* < 0.01). When THI increased, milk fat, protein and lactose percentages decreased, whereas somatic cell count and milk urea nitrogen increased (*P* < 0.05). Evidence indicates that cattle in the Mediterranean region can be exposed to mean THI ≥ 68 for four consecutive months; high environmental temperatures may decrease conception rates by nearly 20% and impair milk production in cows.

## 1 Introduction

Air temperatures are continuously rising worldwide, as assessed by the Intergovernmental Panel on Climate Change (1), which reports a global average temperature increase of approximately 1.0 °C compared to pre-industrial levels (2) and a potential further increase of 1.5 °C by the end of the century (3). In this context, numerous studies report that high environmental temperatures and heat waves (defined as prolonged periods of exceptionally high temperatures that significantly exceed +4 °C to +10.8 °C normal climatic conditions in a given area for 5–10 days), worsened by concomitant high humidity, may impair several physiological functions in dairy cows (4, 5).

Thermoregulation is the physiological process by which mammals maintain their body temperature within a narrow range, despite variations in environmental temperature or their activity. Indeed, cattle are homeothermic and their physiological body temperature ranges between 38 and 39 °C (6), with slight variations depending on environmental conditions and physiological stage of the animal. Specifically, cattle have a thermoneutral zone, defined as the environmental temperature range in which they do not spend additional energy to warm or cool their body, allowing more energy to be diverted toward milk production (7). The thermoneutral zone is considered to range between 4 ° and 25 °C in dairy cattle (7), but this range may be influenced by several factors such as age, breed, and lactation stage (8, 9). For instance, in a cow that weighs 600 kg and produces 40 kg of milk per day with 4% fat, heat production due to metabolic functions accounts for approximately 31% of the energy intake (8, 10). Heifers generate less metabolic heat than adult cows and have a greater body surface area relative to their internal mass, thus they are expected to better withstand high environmental temperatures compared to milking cows (8).

Heat Stress (**HS**) can be defined as the sum of internal and external forces acting on an animal, causing an increase in body temperature and a physiological response (8); in other words, HS refers to an environment that raises the body temperature of cows due to exposure to high temperatures and humidity levels beyond their ability to dissipate heat effectively (11). The potential occurrence of HS in dairy cows is commonly indirectly estimated with the temperature-humidity index (**THI**), which combines ambient temperature and relative humidity (12). Dairy cows exhibit reduced milk production at THI values around 68 (12), whereas heat stress thresholds are not uniform and typically range between 68 and 74, depending on conditions (13). This exposure induces physiological responses aimed at maintaining homeothermy, such as increased respiratory and heart rate, panting, sweating, increased water consumption [i.e. 86.0 vs 81.9 l/day (14)]; such responses may in turn lead to a decrease in feed intake [i.e. 13.6 vs 18.4 kg/day, (14)], milk production [i.e. 16.5 vs 20.0 kg/day, (14)] and fertility, as reproductive efficiency in high-producing dairy cows remains suboptimal and is strongly influenced by metabolic status and early embryonic loss (15). Adequate energy balance and dry matter intake (**DMI**) in the first 4 weeks after calving are crucial for pregnancy success, because cows are typically inseminated, between 70 and 100 days postpartum (15); therefore, reduced feed intake due to high environmental temperatures during the first 3–4 months of lactation may compromise herd fertility. Furthermore, in cows exposed to HS, blood flow is diverted from the udder to the skin to promote body cooling, thus limiting the availability of nutrients for milk synthesis and resulting in decreased milk production and quality (10). These physiological adaptations can reduce milk production by 20–30% during peak lactation (16). Although the impact of HS on reproductive performance and milk production in dairy cattle has been extensively investigated in various regions worldwide, studies conducted in the Mediterranean area remain limited (13).

To mitigate the negative effects of HS on reproductive and productive performance in dairy cows, cooling systems have been developed. High-efficiency mechanical ventilation, often integrated with evaporative cooling technologies, such as misting or sprinkling (17), has been shown to significantly reduce core body temperature and respiration rate during critical heat load periods (18). These systems are typically regulated by automated control units responding to environmental parameters, generally THI, to ensure targeted and energy-efficient activation. The implementation of such systems contributes to mitigating heat-induced declines in feed intake, milk yield, and reproductive efficiency in cows, ultimately supporting the overall health and productivity of the herd (19). Nonetheless, despite the use of automatic cooling systems, high environmental temperatures and humidity may have detrimental impacts on cattle fertility and productivity.

In the present work, we hypothesized that the seasonal variation in environmental temperatures would impair fertility, milk production and composition in Holstein Friesian dairy cattle in two commercial dairy farms in Sardinia (Italy). The aims of this retrospective, longitudinal study were to: (1) investigate the seasonal variation in conception and pregnancy rates, number of services per conception, submission rate, calving to conception interval and gestation length in nulliparous heifers and lactating cows; (2) estimate the seasonal variation in milk production and composition in dairy cows.

## 2 Materials and methods

### 2.1 Farms and cooling systems

This study was conducted in two commercial dairy farms (F1 and F2), located in Sardinia, Italy (F1: 40°5′N 8°3′E; F2: 39°7′48N 8°3′E). F1-herd consisted of 300 lactating cows 270 ± 23 heifers, (mean ± SD) and 249 ± 15 female calves; F2-herd consisted of 500 lactating cows, 413 ± 30 heifers and 447 ± 28 female calves. Cows were milked twice daily and the average (± SD) bulk tank milk yield across the three study years (2021–2023) was 9.13 ± 0.97 ton/day in F1 and 13.51 ± 2.26 ton/day in F2. This corresponded to approximately 30.7 kg/cow/day in F1 and 27.8 kg/cow/day in F2. All animals were housed in free-stall barns with roofs and open side walls; they were fed a total mixed ration (**TMR**) three times per day and had *ad libitum* access to drinking water. Diets were formulated to meet the animals' requirements for energy, protein, mineral, and vitamins based on NRC (2001). The mean chemical composition of the diets was as follows. Dry matter (DM) content on TMR for cows was: F1 = 53.56%, F2 = 51.4% DM for heifers was: F1 = 64.9%, F2 = 55.7%. Crude protein (CP) concentration on TMR offered to cows was 16.74% and 15.8% in F1 and F2, respectively; CP for heifers was 13.53% in F1 and 16.7% in F2. Neutral detergent fiber (NDF) content was 27.36% for F1 cows, 32.2% for F2 cows; 35.86% for F1 heifers and 31.2% for F2 heifers. Acid detergent fiber accounted for 15.13% of TMR for F1 cows, 17.5% for F1 heifers, 17.7% for F2 cows, and 17.4% for F2 heifers. Lignin concentration was 4.3% for F1 cows, 2.6% for F2 cows, 3.1% for F1 heifers and 2.5% for F2 heifers.

In both farms, cows' barns were equipped with a cooling system consisting of fans and showerheads connected to two THI detectors per farm (F1: CMP Impianti S.r.l., Treviso, Italy; F2: Total Dairy Management, TDM, San Paolo, Brescia, Italy). Whenever the THI was greater than 67, the cooling system was automatically activated; ventilation was constant, whereas showerheads were activated at 5-min intervals for 1 min. In both farms the sensors were located along the feeding alley of the cows' barn. In F1 THI monitoring was limited to lactating cows (excluding heifers, dry cows, and post-partum pens); in F2 THI monitoring included lactating cows, dry and postpartum cows (excluding heifers). All detectors were regularly calibrated by trained farm personnel. An operational hysteresis of 3–5 THI units was applied to regulate cooling system activation, ensuring continuous ventilation and intermittent sprinkler cycles until THI decreased by 3–5 units below the activation threshold. In both farms, heifers were located in barns which were not equipped with cooling systems.

### 2.2 Reproductive management and data collection

Reproductive management was performed by a single veterinarian on both farms. Estrus was visually detected twice/day in both heifers and cows; cows were also equipped with the following behavioral monitoring systems: pedometers (TDM, San Paolo, Brescia, Italy) in F1 and sensor collars (DeLaval, Tumba, Sweden) in F2, respectively. Animals detected in estrus before morning milking (0630 h) were artificially inseminated (AI) in the afternoon, whereas heifers/cows detected later in the day were inseminated the following morning (20). Pregnancy was diagnosed via transrectal reproductive ultrasonography (Vetus EQ, Mindray, Medical Sales, Italy equipped with a 5–7 MHZ linear probe) at approximately 30 d (25–36 d) and 60 d (60–66 d) post-AI by the veterinarian.

Reproductive data of sexually mature nulliparous heifers and parous cows recorded from January 2021 to December 2023 in both farms were retrospectively collected and analyzed; 6,655 AI events, 3,282 conceptions and 2,631 parturitions were included in the study. The following reproductive variables were calculated in heifers only: age at first insemination, age at first conception, age at first calving. The following fertility indexes were calculated in both lactating cows and nulliparous heifers: Conception Rate at 30d post-AI (**CR_30d** = number of animals diagnosed as pregnant 30d post-AI/number of inseminated animals), Number of services per conception (**S/C** = number of AIs required to obtain a single pregnancy), Pregnancy Rate at 60d post-AI (**PR_60d** = number of animals diagnosed as pregnant 60d post-AI/number of inseminated animals), monthly Submission rate (**SR** = number of AIs/ number of animals eligible for breeding *per* month), Gestation Length (**GL** = date of calving minus date of successful AI), Calving to Conception Interval (**CCI** = date of conception minus date of calving).

### 2.3 Milk production and data collection

Milk production (**MP**) and milk composition (**MC**) from January 2021 to December 2023 were retrospectively collected from data recorded by the milk-processing company (Cooperativa Produttori Arborea, Società Cooperativa Agricola, Arborea, OR Italy). The total MP was recorded daily, whereas MC was analyzed on a monthly basis. The monthly MC analyses included the following parameters: milk fat percentage (**MFP**), milk protein percentage (**MPP**), milk lactose percentage (**MLP**), somatic cell count (**SCC**), and milk urea nitrogen content (**MUN**). All analyses were performed using standard methods by the dairy company's laboratory.

### 2.4 Estimation of the mean and maximum monthly Temperature Humidity Index

The THI measured in the barns of the two farms by controller devices of the cooling systems were not recorded over the long term and were therefore not available for the retrospective analysis. Thus, climatic data from January 2021 to December 2023 were retrospectively obtained from two meteorological weather stations of the Sardinian Regional Agency for the Environment Protection (Agenzia Regionale per la Protezione dell'Ambiente della Sardegna, ARPAS). The first station was located 5.07 km from F1, and the second was located 5.1 km from F2. Daily minimum and maximum air temperature (Ta, expressed in °C) and relative humidity (RH, expressed as percentage) were used to calculate the mean and maximum monthly temperature humidity index (THI). THI was calculated according to Kibler (21) using the following equation:


$$\begin{array}{l} THI=\left[1.8*Tdb-\left(1-\frac{RH}{100}\right)*\left(Tdb-14.3\right)\right]+32 \end{array}$$ (1)

where *Tdb* is the dry-bulb air temperature (°C) and RH is the relative humidity (%).

### 2.5 Statistical analysis

To evaluate the effect of THI variation on reproductive and milk parameters, data were examined using the following two complementary approaches: (1) a monthly analysis to identify seasonal patterns across 3 years; (2) an analysis based on THI risk categories defined from monthly means at three levels: **Minimum risk** (THI < 56), **Low risk** (56 ≤ THI < 68), and **High risk** (THI≥ 68) applied across the whole period and in both farms. The impact of HS exposure on GL was estimated considering the average THI 60 days prior to calving. All analyses were conducted with R (22). Data were aggregated at the month × year × farm level. Depending on the objective of each analysis, either month (first approach) or the THI category (second approach), together with farm, was included as fixed effects. Year was included as random intercepts. According to the nature of the response variables, linear mixed models (LMM) were fitted for continuous outcomes and generalized linear mixed models (GLMM) were fitted for proportional outcomes using the lme4 package [functions lmer and glmer (23)]. LMM were used for continuous outcomes, GLMM with a binomial distribution and logit link for proportional outcomes, and GLMM with a Gamma distribution and log link for service per conception. Results are reported as least squares means (LSMEANS) on interpretable scales. For non-Gaussian models, LSMEANS were back-transformed to the response scale before reporting. LSMEANS and pairwise comparisons were obtained with the emmeans package (24). Pairwise comparisons with Tukey adjustments were performed and reported only when the overall fixed effect was significant (*P* < 0.05). Associations between the responses and both mean THI and maximum THI were evaluated using Pearson correlations via cor.test from base stats. Results are presented with standard error of the mean. Statistical significance was declared at *P* ≤ 0.05, while 0.05 < *P* ≤ 0.10 was interpreted as indicating a statistical trend

### 2.6 Ethical approval

The study was conducted in accordance with the European Directive 2010/63/EU on the protection of animals used for scientific purposes. Ethical approval was not required because data were retrospectively collected from routine farm management records.

## 3 Results

### 3.1 Annual THI variation

**Figure 1:**
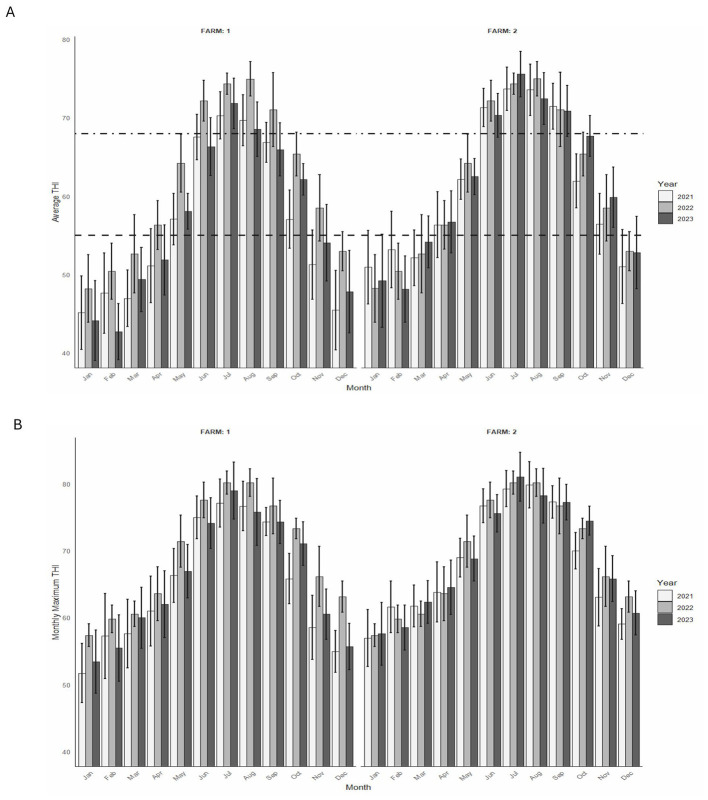
Mean **(A)** and Maximum (**B**; ± SD) monthly temperature-humidity index (THI) recorded by two climatic stations located approximately 5 km away from Farm 1 and 2 from January 2021 to December 2023. Horizontal dashes lines in the top panel indicate the THI thresholds for the risk categories of exposure to heat stress: Minimum risk (THI < 56), Low risk (56 ≤ THI < 68) and High risk (THI≥ 68).

During the 3 years, the average monthly THI was 56.46 ± 1.63 (range 42.70 to 71.82) and 61.53 ± 1.54 (range 48.09 to 75.57) obtained from two meteorological weather stations nearby F1 and F2, respectively (Figure 1A). Over the 3 years, the mean maximum THI recorded per month in F1 was 65.29 ± 1.47 (range 51.68 to 78.97) and 68.68 ± 1.35 in F2 (range 56.95 up to 81.02; Figure 1B). The mean monthly THI consistently showed a seasonal pattern during each year (Figure 1A). Minimum risk conditions (monthly THI < 56) regularly occurred in January, February, March and December in both farms. Low risk conditions (56 ≤ THI < 68) characterized the months of April, May, October and November in both farms. For instance, in April, the mean THI was 57.1 ± 0.43 in F1 and 56.4 ± 0.39 in F2. From June to September, both farms experienced High risk conditions (THI ≥ 68).

### 3.2 Fertility indexes in non-lactating heifers

Over the three-year period, the following mean fertility indexes were reported in nulliparous heifers: CR_30d = 57% (range 50 – 64%), PR_60d = 53% (range 48−57%), S/C = 1.86 (range 1.59−2.19), monthly SR = 12.73% (range 9.39−17.88%), GL = 273.71 d (range 271.16−275.03 d), age at first insemination = 16.2 mo. (range 15.56−17.02 mo.), age at first conception = 16.6 mo. (range 15.77−17.11 mo.), age at first calving = 25.9 mo. (range 25.24−26.95 mo.). In heifers, most of the fertility parameters were constant throughout the year, except for monthly SR, which was influenced by month (*P* < 0.001; Table 1); specifically, SR reached its maximum in May (17.88%) and its minimum in September (9.39%; *P* < 0.05). Gestation length was not influenced by the mean THI registered in the 2 months prior to calving (data not shown). Fertility indexes were similar among years in heifers.

**Table 1:** Monthly fertility indexes (mean) in nulliparous heifers from January 2021 to December 2023.

Parameter	CR_30d (%)	PR_60d (%)	S/C (*n*)	SR (%)	Age at first insemination (mo.)	Age at first conception (mo.)	Age at first calving (mo.)
Jan	57	52	1.87	11.59^ab^	15.65	16.30	25.39
Feb	59	57	1.79	12.32^ab^	16.06	16.54	25.60
Mar	57	54	1.88	15.19^ac^	16.10	16.60	25.71
Apr	58	55	1.65	13.09^abc^	15.88	16.78	25.94
May	58	54	1.87	17.88^c^	16.05	16.92	26.07
Jun	58	56	1.69	10.85^ab^	15.56	16.81	25.96
Jul	56	53	1.85	10.65^ab^	16.43	16.58	25.24
Aug	51	49	2.11	11.55^ab^	15.63	15.77	25.75
Sep	50	48	2.19	9.39^b^	16.55	16.52	26.14
Oct	64	57	1.59	13.99^abc^	17.02	17.11	25.96
Nov	59	55	2.11	14.16^ac^	16.66	16.84	26.95
Dec	59	55	1.78	12.08^ab^	16.38	16.52	26.57
SEM	0.05	0.04	0.16	1.34	0.36	0.41	0.38
*P*–value	0.765	0.973	0.122	< 0.001	0.196	0.542	0.085

CR_30d: Conception Rate at 30 days post-AI; PR_60d: Pregnancy Rate at 60 days post-AI; S/C: Number of services per conception; SR (%): Number of AIs/ number of animals eligible for breeding per month.

^a, b, c^ Different superscripted letters within the same column indicate statistical differences (P < 0.05).

When analyzed according to THI risk categories, monthly SR in heifers varied among categories (*P* < 0.001; Table 2); it was higher in Minimum and Low risk conditions compared to the High-risk category. The other fertility indexes were similar across the three THI categories in heifers (Table 2).

**Table 2:** Least squares mean reproductive parameters in non-lactating heifers according to the THI mean category effect.

Parameter	Minimum risk (THI < 56)	Low risk (56 ≤ THI < 68)	High risk (THI ≥68)	SEM	*P*-value
CR_30d (%)	59	59	53	0.02	0.219
PR_60d (%)	55	54	51	0.02	0.415
S/C (n)	1.79	1.91	1.92	0.11	0.434
SR (%)	10.75^a^	14.14^b^	12.93^b^	0.99	< 0.001
GL (d)	273.79	274.15	273.41	0.824	0.666
Age at first insemination (mo.)	16.14	16.26	16.08	0.240	0.775
Age at first conception (mo.)	16.57	16.84	16.39	0.27	0.246
Age at **first** calving (mo.)	26.02	26.04	25.69	0.23	0.406

CR_30d: Conception Rate at 30 days post-AI; PR_60d: Pregnancy Rate at 60 days post-AI; S/C: Number of services per conception; SR (%): Number of AIs/ number of animals eligible for breeding per month; GL: Gestation length.

^a, b^ Different superscripted letters within the same row indicate statistical differences (P < 0.05).

In heifers, age at first insemination, first conception and first calving were negatively correlated with the mean monthly THI (*P* < 0.05; Table 3). CR_30d, PR_60d, monthly SR, S/C and GL were not correlated with the mean and maximum THI (Table 3). No significant correlation was observed between the fertility parameters and THI-risk classes in heifers (data not shown).

**Table 3:** Pearson correlations between mean monthly and maximum monthly temperature humidity index (THI) and fertility indexes in heifers.

Parameter	Mean monthly THI		Maximum monthly THI	
	*r*	*P*-value	*R*	*P*-value
CR_30d	−0.041	0.74	−0.03	0.80
PR_60d	−0.013	0.91	−0.009	0.94
S/C	0.041	0.73	0.029	0.81
SR (%)	−0.044	0.712	−0.056	0.643
GL	0.116	0.331	0.102	0.393
Age at first insemination	−0.196	0.01	−0.120	0.32
Age at first conception	−0.243	0.04	−0.166	0.16
Age at **first** calving	−0.276	0.02	−0.223	0.06

CR_30d: Conception Rate at 30 days post-AI; PR_60d: Pregnancy Rate at 60 days post-AI; S/C: Number of services per conception; SR (%): Number of AIs/ number of animals eligible for breeding per month; GL: Gestation length.

### 3.3 Seasonal fertility variation in lactating cows

Over the three-year period, the mean fertility indexes in lactating cows were: CR_30d = 47.76% (range 32.95–59.61%), PR_60d = 37.05% (range 22.14–52.18%), S/C = 2.18 (range 1.79–3.11), monthly SR = 16.64% (range 11.31–21.42%), CCI = 151.24 d (range 138.48–168.37 d), GL = 276.5 d (range 274.9–278.31 d).

**Figure 2:**
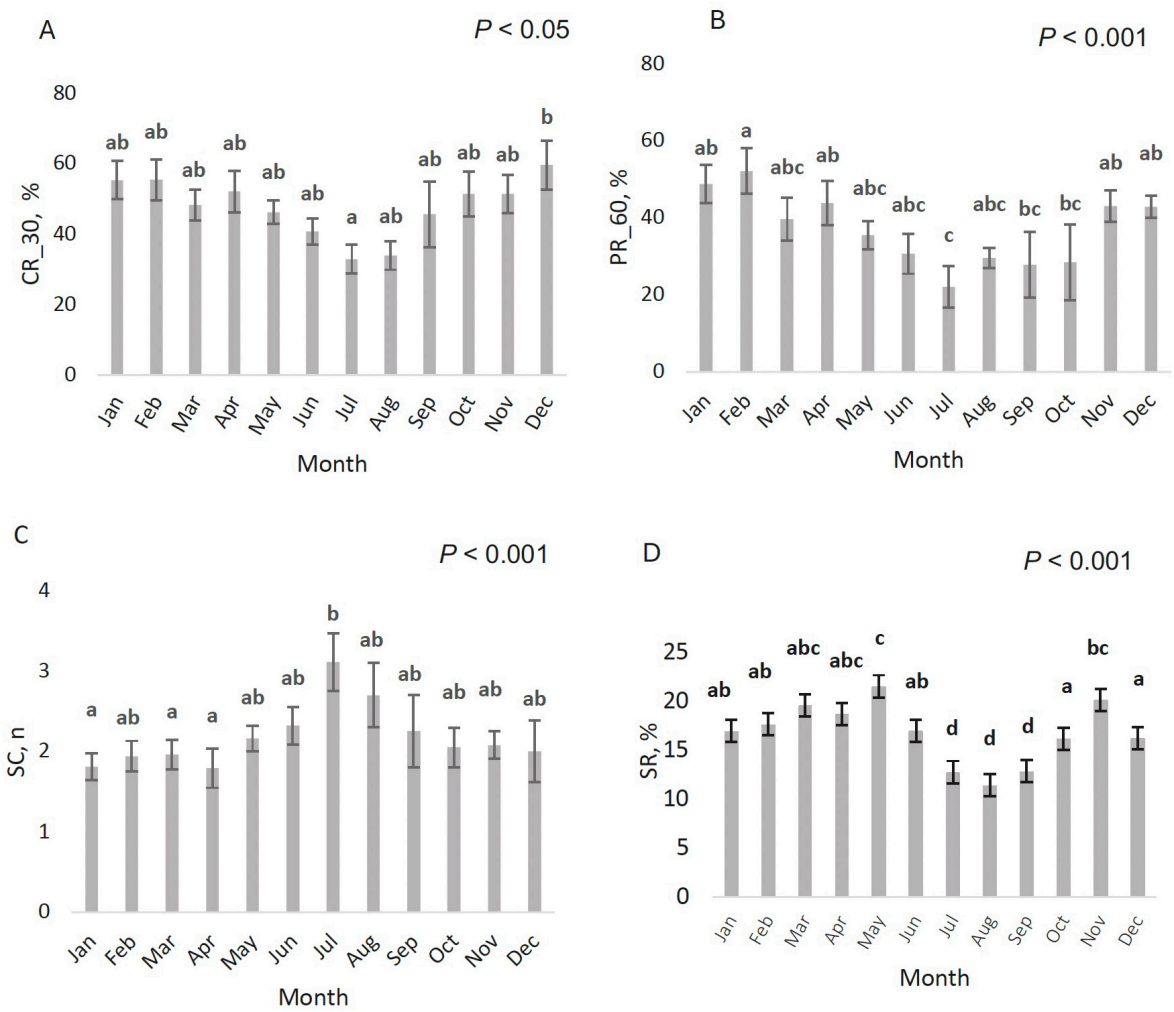
Monthly variation (mean ± SEM) in reproductive parameters in cows from January 2021 to December 2023. **(A)** Conception rate at 30 d post-AI (CR_30d, %); **(B)** Pregnancy rate at 60 d post-AI (PR_60d, %); **(C)** Services per conception (n); **(D)** Submission rate (SR, %): Number of AIs/ number of animals eligible for breeding *per* month (*n*). Least squares means with different letters (a, b, c) differ significantly (*P* < 0.05).

Conception Rate at 30d post-AI, Pregnancy rate at 60d post-AI, S/C and monthly SR were significantly influenced by month (CR_30d: *P* < 0.05; PR_60d, S/C and monthly SR: *P* < 0.001; Figure 2). Specifically, the highest CR_30d was observed in December (59.61%), whereas the lowest values were reported in July (32.95%; *P* < 0.05; Figure 2A). Similarly, PR_60d reached its maximum in February (52.18%), progressively decreased until July (22.14%) and increased from August to December (*P* < 0.05; Figure 2B). The highest S/C was recorded in July (3.11) and the lowest in January (1.81), March (1.96) and April (1.79, *P* < 0.05; Figure 2C). Monthly SR peaked in May (21.42%) and November (20,04%) compared to July, August and September (12.64%, 11.31%, 12,76%; *P* < 0.05; Figure 2D). Finally, CCI did not show significant differences among months, and GL was not influenced by the mean THI registered in the last 2 months of gestation.

When analyzed according to the THI risk categories, CR_30d, PR_60d, S/C and SR were different among categories (*P* < 0.001; Table 4). The mean CR_30d was higher in the Minimum risk (55.16%) compared to the High risk (37.20%) category (*P* < 0.05). Similarly, PR_60d was higher in the Minimum risk category (45.75%) compared to the Low and High-risk groups (33.97 and 29.43%, respectively). More S/C were required in the High-risk months (2.85, *P* < 0.05) compared to Minimum and Low risk categories (1.87, and 2.08 *P* < 0.05, respectively). Monthly SR was lower in High risk compared to both the Low and Minimum risk categories (*P* < 0.05). On the other hand, GL and CCI were similar among risk categories.

**Table 4:** Least squares mean reproductive parameters in cows according to the temperature-humidity index (THI) mean category effect.

Parameter	Minimum risk (THI < 56)	Low risk (56 ≤ THI < 68)	High risk (THI ≥68)	SEM	*P*-value
CR_30d (%)	55.16^b^	47.76^b^	37.20^a^	0.028	< 0.001
PR_60d (%)	45.75^b^	33.97^a^	29.43^a^	0.026	< 0.001
S/C (n)	1.87^b^	2.08^b^	2.85^a^	0.20	< 0.001
SR (%)	17.89^b^	17.95^b^	13.11^a^	1.10	< 0.001
GL (n)	276.47	276.58	276.98	0.45	0.660
CCI (d)	147.11	156.33	153.45	5.54	0.185

CR_30d: Conception Rate at 30 days post-AI; PR_60d: Pregnancy Rate at 60 days post-AI; S/C: Number of services per conception; SR (%): Number of AIs/ number of animals eligible for breeding per month; GL: Gestation length; CCI: Calving to Conception Interval.

^a, b^ Least squares mean with different superscript letters are significantly different for each parameter (P < 0.05).

In cows, CR_30d and PR_60d were negatively correlated with both mean and maximum monthly THI (*P* < 0.001), whereas S/C was positively correlated with the mean and maximum monthly THI (*P* < 0.001; Table 5). Calving to conception interval, monthly SR and GL were not correlated with THI.

**Table 5:** Correlation analysis between reproductive parameters and mean monthly and maximum monthly temperature humidity index (THI) in dairy cows.

Parameter	Mean monthly THI		Maximum monthly THI	
	R	*P*-value	r	*P*-value
CR_30d	−0.521	< 0.001	−0.511	< 0.001
PR_60d	−0.570	< 0.001	−0.565	< 0.001
S/C	0.569	< 0.001	0.557	< 0.001
SR (%)	−0.141	0.237	−0.163	0.170
GL	0.016	0.893	0.040	0.737
CCI	0.088	0.464	0.124	0.299

CR_30d: Conception Rate at 30 days post-AI; PR_60d: Pregnancy Rate at 60 days post-AI; S/C: Number of services per conception; SR (%): Number of AIs/ number of animals eligible for breeding per month; GL: Gestation length; CCI: Calving to Conception Interval.

### 3.4 Influence of season on milk production and composition

The mean MFP and MPP were 4.02% and 3.37%, respectively, varying between 3.88–4.2 % and 3.30–3.49%. The average MLP was 4.77%, with a range of 4.72–4.85%. The mean SCC reached 259.95 × 103/ml and fluctuated from 235.37 to 288.28 × 103/ml. Finally, MUN averaged 21.37 mg/dl, ranging between 18.37 and 23.81 mg/dl.

**Figure 3:**
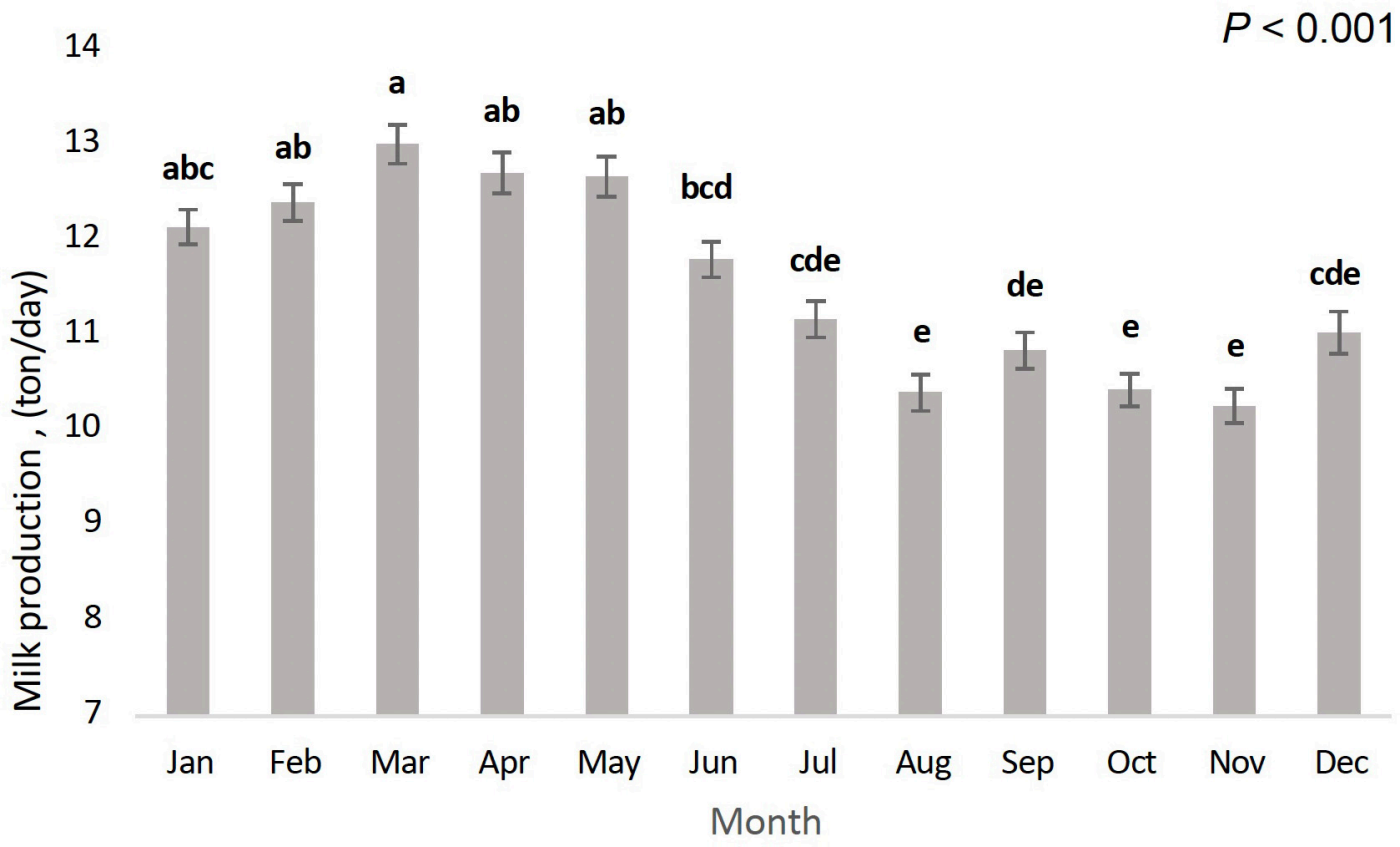
Monthly variation (mean ± SEM) in milk production (ton/day) from the year 2021 to 2023. Least squares means with different letters (a, b, c, d, e) differ significantly (*P* < 0.05).

The mean daily MP varied among months (*P* < 0.001; Figure 3), with the highest production in March (13.01 tons/day; *P* < 0.05) and the lowest yields in August, October and November (10.4; 10.43; 10.26 tons/day; *P* < 0.05). All the indexes of milk compositions were influenced by month (MFP, MUN *P* < 0.001; MLP, SCC *P* < 0.005), except for MPP which tended to change among months (*P* = 0.069; Table 6). Milk fat percentage was highest in December (4.20%) and lowest in June and July (< 3.9%; *P* < 0.05). Milk lactose percentage was higher in December (4.85 %, *P* < 0.05) compared to the months from August to November ( ≤ 4.75%; *P* < 0.05). An opposite trend was observed in SCC, with maximum values in September (288.07 × 103 cells/ml) and minimum values in April (231.88 × 103 cells/ml; *P* < 0.05). Finally, MUN concentrations were higher in May, June and July compared to March (*P* < 0.05).

**Table 6:** Least squares mean of milk composition according to the month.

Parameter	MFP %	MPP %	MLP %	SCC × 103/ml	MUN (mg/dl)
Jan	4.11^abc^	3.38	4.79^ab^	260.81^abcd^	19.67^ab^
Feb	4.10^abc^	3.34	4.79^ab^	234.14^abc^	18.80^ab^
Mar	4.03^abcde^	3.35	4.80^ab^	254.60^abcd^	18.37^a^
Apr	3.99^abde^	3.37	4.80^ab^	231.88^a^	20.18^abc^
May	3.93^abde^	3.33	4.79^ab^	239.49^abcd^	22.82^bc^
Jun	3.88^d^	3.30	4.79^ab^	265.80^abcd^	23.81^c^
Jul	3.88^d^	3.31	4.78^ab^	283.57^bd^	23.01^bc^
Aug	3.90^d^	3.36	4.75^a^	281.61^bcd^	21.46^abc^
Sep	4.00^abde^	3.43	4.74^a^	288.07^d^	22.16^abc^
Oct	4.08^abce^	3.46	4.72^a^	258.91^abcd^	22.13^abc^
Nov	4.15^bc^	3.49	4.73^a^	239.74^abcd^	20.98^abc^
Dec	4.20^c^	3.43	4.85^b^	231.47^ac^	23.05^bc^
SEM	0.050	0.03	0.012	18.74	0.012
*P*–value	< 0.001	0.069	0.002	0.002	< 0.001

MFP: milk fat percentage; MPP: milk protein percentage; MLP: milk lactose percentage; SCC: somatic cells count; MUN: milk urea nitrogen.

^a, b, c, d, e^ Least squares means with different superscript letters are significantly different within column (P < 0.05).

Milk production and MUN were not correlated with mean and maximum monthly THI (Table 7). Milk fat percentage, MPP and MLP were negatively correlated with the mean THI (*P* < 0.05) and maximum THI (*P* < 0.05; Table 7). On the opposite, SCC was positively correlated with mean and maximum THI (*P* < 0.05).

**Table 7:** Pearson correlations between mean and maximum monthly temperature humidity index (THI) and milk production and composition parameters.

Parameter	Mean monthly THI		Maximum monthly THI	
	*R*	*P*-value	*r*	*P*-value
MP	0.094	0.434	0.033	0.781
MFP	−0.539	0.000	−0.482	0.000
MPP	−0.300	0.010	−0.256	0.030
MLP	−0.368	0.001	−0.318	0.006
SCC	0.235	0.047	0.239	0.043
MUN	0.098	0.413	0.143	0.231

MP, Milk production; MFP, milk fat percentage; MPP, milk protein percentage; MLP, milk lactose percentage; SCC, somatic cells count; MUN, milk urea nitrogen.

Milk production and composition differed among THI categories (Table 8). MP was enhanced in the Minimum risk category (11.91 ton/day) compared to the High risk class (10.90 ton/day; *P* < 0.01). Milk fat percentage was higher in the Minimum risk compared to both Low and High risk categories (*P* < 0.001), whereas MPP was greater in Low vs High risk category (3.40 vs 3.34%; *P* < 0.05). Conversely, SCC was higher in the High risk months compared to both the Low and Minimum risk ones (*P* < 0.001); MUN was impaired in Minimum risk vs Low and High risk conditions (*P* < 0.01).

**Table 8:** Least squares means of milk production and composition according to the THI mean category effect.

Parameter	Minimum risk (< 56)	Low risk (56–68)	High risk (≥68)	SEM	*P*–value
MP (ton/day)	11.91^b^	11.52^ab^	10.90^a^	0.153	0.008
MFP %	4.10^b^	3.99^a^	3.93^a^	0.03	< 0.001
MPP %	3.39^ab^	3.40^b^	3.34^a^	0.009	0.020
MLP %	4.79^b^	4.76^ab^	4.75^a^	0.007	0.030
SCC × 103/ml	245.88^b^	248.80^b^	277.49^a^	0.087	< 0.001
MUN mg/dl	20.42^b^	21.83^ab^	22.72^a^	0.009	0.004

MP, Milk production; MFP, milk fat percentage; MPP, milk protein percentage; MLP, milk lactose percentage; SCC, somatic cells count; MUN, milk urea nitrogen.

^a, b^ Least squares means with different superscript letters are significantly different for each parameter (P < 0.05).

## 4 Discussion

The present study provides novel evidence to heat stress on fertility and milk production in dairy cattle bred in the Mediterranean area. The most significant findings are that: (1) on average, cattle were exposed to mean THI≥ 68 (high risk of heat stress) for four consecutive months per year and, similarly, to mean THI ranging from 56 to 68 (Low risk) for 4 months/ year, indicating the relevance of exposure to heat stress in the region; (2) in heifers, most fertility indexes were constant during the year, yet submission rate following visual detection of natural heat decreased when THI ≥68; (3) in lactating cows, when THI exceeded 68, conception rate 30 days post service and pregnancy rate 60 days post AI decreased by approximately 18 and 16 percentage points, respectively, compared to mean THI < 56 (Minimum risk of heat stress), respectively; (4) the submission rate was lower under High risk compared to Minimum risk conditions in cows; (5) in cows, the submission rate declined in summer months and was severely reduced in High risk months compared to both Low and Minimum risk periods; (6) milk production and composition changed monthly and were impaired when High risk conditions occurred.

The analysis of monthly THI revealed a constant seasonal pattern during the 3 years of the study, with prolonged periods of high thermal load. Mean THI exceeded 68 from June to September in both farms, indicating a prolonged exposure to conditions leading to heat stress (12). In addition, the maximum THI recorded per month was constantly higher than 70 from June to September, with peaks of 79 and 81. Such environmental conditions were slightly warmer than those we previously reported in the same region of the island, as the average monthly THI was 69 and 70 from May to August 2015 and 2016, respectively, with peaks of 75 (25). These ranges indicate that cows in the area presently face high risk of heat stress in hot months and suggest that cattle were exposed to sustained and cumulative thermal stress for four consecutive months. It should be noted that, in both the present and the previous study we conducted in the area (25), THI data were derived from regional meteorological stations located outside the farms. This was due to the fact that the commercial THI sensors installed on the farms did not retain temperature and humidity records suitable for a three-year retrospective analysis. It is plausible that THI within the barn may have differed from that recorded by the meteorological stations; indeed, an investigation conducted in Germany provided evidence of higher THI in the barns compared to the official meteorological station (26), therefore, further studies evaluating the microclimate under on-farm conditions are needed to improve the accuracy of exposure assessment of cattle in confined systems in the region. Nevertheless, regional meteorological stations undergo regular technical validation, and their data are continuously monitored, ensuring high reliability of the recorded measurements and these data have been employed for other investigations on heat stress in dairy cattle in Italy (27, 28). Thus, we consider these data to be robust indicators of the potential risk of exposure to high environmental temperature and humidity. Indeed, prolonged exposure to elevated THI is increasingly recognized as the primary driver of long-term impairments in productivity and fertility in cattle (4, 5, 29); such exposure likely induces chronic physiological strain that cannot be completely mitigated by cooling systems activated at fixed thresholds, potentially. Climate projections report a tendency for warmer and drier Mediterranean climate (30) and the Mediterranean Sea area has been identified as a worldwide “hot spot” for its vulnerability to climate change (31). Consequently, the risk of dairy cattle being exposed to heat stress in the Mediterranean basin is likely to increase in the next foreseeable future. Based on these notions, investigations on the prevalence of heat stress exposure and on the efficacy of mitigating systems, potentially with specific data of the farm microclimate, in the Mediterranean area may be valuable for the dairy industry.

The THI is determined by combining ambient temperature and relative humidity and is widely used on farm as an environmental-based indicator to indirectly estimate the heat load in cattle; cows are usually considered to be suffering from HS when exposed to THI of 68 or higher (12); yet other factors may influence such threshold. Evidence indicates that in lactating cows respiratory rate increases when THI is higher than 65 if cows are lying and 70 if cows are standing, respectively; heart rate and rectal temperature increase when THI exceeds 72 and 70, respectively (32). Also, THI thresholds ranging from 68 to 72 are associated with significant impairment of milk yield in subtropical climates (33), but in temperate and continental climates a decrease in milk production has been reported with THI between 60 and 65 (34, 35). In the present study, reproductive and milk data were analyzed both monthly and using THI risk categories; these complementary approaches were chosen to investigate the potential impairment in fertility and milk production concurrent with THI increase both prior and after the threshold (THI = 68) which activated cooling systems for cows in both enrolled farms. Overall, this dual analytical approach allowed us to evaluate not only the effects of acute heat stress episodes, but also the impact of sustained sub-threshold thermal load. As previously mentioned, the mean monthly THI was calculated from meteorological data recorded outside the barn; therefore, potential climatic differences between the barn environment and the analyzed data cannot be ruled out and should be considered when interpreting these findings.

Heifers were overall more fertile compared to lactating cows; mean conception rate at 30 days post-AI and pregnancy rate at 60 days post-AI were approximately 10% and 16% higher in heifers than cows, respectively. Moreover, conception rate, pregnancy rate and number of services per conception remained stable across months and THI risk classes in heifers. Nonetheless, the lowest monthly conception rate occurred from July to September, suggesting a potential negative impact of high environmental temperatures on fertility. Also, during summer and in High risk months, a significant decline in the submission rate was observed, reaching the minimum in September. In both farms enrolled in the study, artificial insemination in heifers was performed following visual detection of natural estrus, because automated activity sensors or pedometers were not used in nulliparous animals, as a result of a farm management decision. Also, cooling systems were not used in heifers in the present study, thus the impact of high temperatures was not mitigated. We speculate that high temperatures lead to a decrease in the length and intensity of estrous behavior, impairing heat detection and decreasing the number of services performed. Estrous behavior in heifers is not influenced by factors such as milk production and variable metabolic states, which are known to influence estrus expression in lactating dairy cows (36). Nonetheless, evidence indicates that hot climatic conditions reduce estrus length in heifers; under spring and natural summer climatic conditions the average duration of estrus was 20 and 14 h, respectively, and the incidence of clinical anestrus was 33% among the heifers during the hot period (37). More recently an investigation conducted with leg mounted accelerometers in heifers reported that estrus relative increase in activity was greater for episodes that happened during the winter than during the spring and the summer (38). Also, during the warm season, the day of estrus had lower frequency of standing bouts and the longest standing bout of the day of heat was around 25% shorter than during the cold season (39). In the present study, the timing of service in heifers was based on the sole visual detection of standing to be mounted behavior. It is plausible that the observed decline in the total number of monthly artificial inseminations in summer may have resulted from poor intensity of expression linked to hyperthermia or insufficient observation by the farmers or both. Without automated activity monitoring in heifers, it is not possible to definitively disentangle the decline in services from potential biases related to observer fatigue or logistical constraints during extreme summer heat. On the other hand, the age at first AI, conception and calving did not vary across months or THI classes in heifers. Overall, these results suggest that, under the conditions of the present study, heat stress did not substantially impair fertility in heifers; however, these findings may have been influenced by the sole use of visual estrus detection in heifers. A study aimed at investigating seasonal variations in fertility in heifers in the Mediterranean area using automated devices for detection of estrus would be warranted.

Lactating cows exhibited pronounced seasonal variation in fertility parameters. Conception rate at 30d post-AI declined by approximately 27% from December to July in the present study; such decrease is higher than the 15% reported in the South of Italy (40) and higher than the 23% decrease in conception rate reported in Northern Spain (41), but similar to the 20 to 30% decline observed in the subtropical area (42), suggesting that the impact of heat stress on bovine fertility in the Mediterranean is severe. Also, previous evidence indicates that conception rates decline when cows are exposed to high heat load not only on the day of service, but also in week 5, 3 and 1 prior to service, as well as 6 days after insemination (43). This may explain the minimum in conception rates that we report in July and August, which were preceded by hot weather in June; exposure to high mean THI for several consecutive weeks may have contributed to the notable decline in conception rate observed in July and August. Similarly, cows diagnosed pregnant 60d post-AI were almost halved when insemination was performed in July compared to February.

Conception rate at 30d post-AI and pregnancy rate at 60d post-AI were highest when mean THI was lower than 56 (CR_30d 55.16% and PR_60d 45.75%) and decreased progressively in the Low risk category, reaching their lowest values in the High risk category (CR_30d 37.20%, PR_60d 29.43%). Conception rate decreased by approximately 7% between the Minimum risk and Low risk THI classes, indicating that impairment of reproductive performance may occur under moderate heat load (THI 56–68), when automatic cooling systems are not active. Also, despite the activation of the cooling systems, when the mean monthly THI exceeded 68 conception rates declined by approximately 18%; this finding suggests that heat-related fertility impairment may still occur even when cooling strategies are implemented.

The higher number of services per conception observed during the summer months, the strong negative correlation between THI and fertility outcomes as well as the positive association with S/C confirm the high thermal sensitivity of reproductive function in lactating cows. The reduction in submission rate observed in the present study during summer was followed by a gradual recovery in autumn, a pattern typical of herds managed without the routine use of estrus synchronization protocols (44). This trend likely reflects a decrease in estrus expression and detection induced by heat stress. Evidence indicates that environmental hyperthermia reduces estradiol secretion and decreases both the duration and intensity of estrus behavior in lactating cows, leading to silent ovulation and consequently fewer cows being inseminated following detection of natural heat (44, 45).

In the present study, the calving-to-conception interval did not show a monthly or clear seasonal pattern. This may be explained by the fact that the calving to conception interval is strongly influenced by management and breeding strategies. Moreover, inconsistent effects of heat stress (and heat abatement strategies) have been reported on the calving to conception interval and days open (46). Similarly, gestation length was not affected by THI in the 60 days prior to calving or by THI category in both heifers and cows. In contrast, cows exposed to summer heat stress in Florida (average THI 77–79) during the final two months of gestation exhibited a reduction in gestation length of approximately three days compared with herd mates receiving active cooling (47). Similarly, dry cows exposed to average THI 78 from approximately 46 days prior to calving had a four-day shorter gestation length compared with cooled cows (48). Gestation length was approximately four days longer in cows housed in pens with active cooling systems compared to cows in pens that only received shade with THI constantly exceeding 68 at day and night (49). On the other hand, the analysis of data from seven Florida dairy farms, including parturitions over a 52-yr period (1923 to 1974) reported no difference in gestation length between warm and cool seasons in Florida (50). In our dataset, the lack of effect of heat stress two months before calving on gestation length may be explained by the presence of unaccounted confounding variables that potentially obscured the influence of high environmental temperatures. For instance, calving age of the dam, milk yield, twinning and calf sex affected gestation length in cattle (51).

Milk production decreased markedly when THI exceeded 68 compared with thermoneutral conditions and the resulting monthly variation in bulk milk production followed the typical Mediterranean production pattern, characterized by a spring peak, summer depression, and partial autumn recovery (13, 52, 53). However, no direct association was detected between monthly bulk milk production and THI. This apparent discrepancy is potentially explained by scale mismatch: heat stress affects milk secretion at short time scales, whereas monthly aggregation smooths short thermal peaks responsible for production losses. In addition, bulk tank milk represents the composite response of cows at different lactation stages, attenuating individual variability. Previous studies have shown that the milk production – THI relationship is evident in daily records, but markedly reduced after temporal aggregation (12, 54). Furthermore, milk production exhibits a carry-over effect, as recovery from exposure to heat stress requires several days or weeks due to persistent metabolic and mammary alterations (8, 53).

Milk fat, protein and lactose percentages decreased during high THI periods, indicating negative correlations with elevated environmental temperatures. This is in agreement with previous evidence from Northern Italy reporting that milk yield and composition were significantly affected by average daily THI during summer months (27). More, heat exposure was associated with elevated milk urea nitrogen and somatic cell count. Although the potential causes of these variations were not investigated in the present study, we may speculate that factors such as reduced DMI, negative energy balance, altered rumen fermentation and reduced blood flow to the mammary gland may have contributed to these changes (52). Overall, the results indicate that heat stress induces prolonged metabolic and mammary adaptations that extend beyond short thermal peaks, resulting in a persistent seasonal decline in milk production and quality that remains evident even when daily bulk milk data are averaged at the monthly level.

Due to the absence of physiological biomarkers, such as hormonal, inflammatory, or oxidative stress indicators, our study does not contribute to the in-depth mechanistic understanding of responses to heat stress in dairy heifers and cows. Also, use of aggregated milk production data does not allow for the identification of individual variability factors or the characterization of differences in heat tolerance between individuals. Similarly, analyses of reproductive data were conducted on data aggregated at the month × year × farm level, which limits inferences at the individual level and constraints causal interpretation. In addition, since THI data measured in the barn were not available, climatic data from two meteorological weather stations located approximately 5 km from the farms were included in the analysis. This may have reduced the accuracy of the characterization of high THI exposure and is acknowledged as a limitation of the study. Nonetheless, the present investigation provides novel knowledge on the incidence of exposure to high environmental temperatures in dairy cattle in the Mediterranean area.

## 5 Conclusions

Overall, this study indicates that prolonged exposure to high heat stress (THI ≥ 68 for ~4 months/year) represents a constraint on reproductive and productive performance in dairy cattle in the Mediterranean area. Lactating cows were particularly vulnerable, with reduction in conception rate at 30 days post insemination and pregnancy rates at 60 days post insemination, together with an increase in number of services per conception even under moderate risk conditions (THI 56–68), whereas heifers showed greater resilience. The summer decrease in submission rate also suggests potential difficulties in estrus detection under high environmental temperatures, due to changes in management and/or poor estrus expression. These findings highlight the need to prioritize improved heat detection and targeted breeding management during hot months. Milk production and quality were compromised during and after periods of high THI, likely indicating cumulative effects of heat stress exposure rather than acute responses. It may be speculated that high environmental temperatures were associated with impaired fertility and productivity in cows, despite the use of cooling strategies. Results support the need for integrated mitigation strategies (such as more effective cooling, targeted nutrition, reproductive management, and selection of tolerant phenotypes) to ensure adequate fertility and productivity.

## Data Availability

The raw data supporting the conclusions of this article will be made available by the authors, without undue reservation.
